# BoHV-4-Based Vector Single Heterologous Antigen Delivery Protects STAT1^(-/-)^ Mice from Monkeypoxvirus Lethal Challenge

**DOI:** 10.1371/journal.pntd.0003850

**Published:** 2015-06-18

**Authors:** Valentina Franceschi, Scott Parker, Sarah Jacca, Ryan W. Crump, Konstantin Doronin, Edguardo Hembrador, Daniela Pompilio, Giulia Tebaldi, Ryan D. Estep, Scott W. Wong, Mark R. Buller, Gaetano Donofrio

**Affiliations:** 1 Department of Medical-Veterinary Science, University of Parma, Parma, Italy; 2 Molecular Microbiology and Immunology, Saint Louis University School of Medicine, St. Louis, Missouri, United States of America; 3 Vaccine and Gene Therapy Institute, Oregon Health and Science University, Beaverton, Oregon, United States of America; The George Washington University School of Medicine and Health Sciences, UNITED STATES

## Abstract

Monkeypox virus (MPXV) is the etiological agent of human (MPX). It is an emerging orthopoxvirus zoonosis in the tropical rain forest of Africa and is endemic in the Congo-basin and sporadic in West Africa; it remains a tropical neglected disease of persons in impoverished rural areas. Interaction of the human population with wildlife increases human infection with MPX virus (MPXV), and infection from human to human is possible. Smallpox vaccination provides good cross-protection against MPX; however, the vaccination campaign ended in Africa in 1980, meaning that a large proportion of the population is currently unprotected against MPXV infection. Disease control hinges on deterring zoonotic exposure to the virus and, barring that, interrupting person-to-person spread. However, there are no FDA-approved therapies against MPX, and current vaccines are limited due to safety concerns. For this reason, new studies on pathogenesis, prophylaxis and therapeutics are still of great interest, not only for the scientific community but also for the governments concerned that MPXV could be used as a bioterror agent. In the present study, a new vaccination strategy approach based on three recombinant bovine herpesvirus 4 (BoHV-4) vectors, each expressing different MPXV glycoproteins, A29L, M1R and B6R were investigated in terms of protection from a lethal MPXV challenge in STAT1 knockout mice. BoHV-4-A-CMV-A29LgD_106_ΔTK, BoHV-4-A-EF1α-M1RgD_106_ΔTK and BoHV-4-A-EF1α-B6RgD_106_ΔTK were successfully constructed by recombineering, and their capacity to express their transgene was demonstrated. A small challenge study was performed, and all three recombinant BoHV-4 appeared safe (no weight-loss or obvious adverse events) following intraperitoneal administration. Further, BoHV-4-A-EF1α-M1RgD_106_ΔTK alone or in combination with BoHV-4-A-CMV-A29LgD_106_ΔTK and BoHV-4-A-EF1α-B6RgD_106_ΔTK, was shown to be able to protect, 100% alone and 80% in combination, STAT1^(-/-)^ mice against mortality and morbidity. This work demonstrated the efficacy of BoHV-4 based vectors and the use of BoHV-4 as a vaccine-vector platform.

## Author Summary

Human Monkeypox is a zoonotic neglected tropical disease, and the majority of human infections occur in the poor rural areas of central Africa. Human Monkeypox was discovered when the Smallpox vaccination campaign stopped, exposing the immunologically unprotected population to the risk of Monkeypoxvirus infection. Currently, no safe and efficacious vaccines for prevention of Monkeypox are available for an epidemic setting, and further studies are needed for the development of a safe and efficacious vaccine. In this study, safety and protection of three recombinant Bovine Herpesvirus 4-based vectors, each delivering a Monkeypox glycoprotein, was evaluated in a suitable mouse model. All three recombinants appeared safe, and one of them, delivering a single dose of Monkeypox M1R glycoprotein, protected 60% of mice against a lethal dose of Monkeypoxvirus; when given as a prime/boost, it afforded 100% protection. Although further investigation into other Monkeypox models, including non-human primate, is needed to assess bovine herpesvirus 4-based vector delivering M1R safety and protection, the findings of this work pave the way for the potential use of Bovine Herpesvirus 4-based vectors for other category A agents.

## Introduction

Monkeypox virus (MPXV) is an orthopoxvirus with a broad-host range capable of infecting many animal species [1]. In humans, MPXV causes a disease very similar to the closely related variola virus, the etiological agent of smallpox: a rash (a 2–4 week period where macules develop and form papules, vesicles and pustules) which is preceded by a 10–14 day incubation, followed by a 1–3 day interval characterized by a prodromal fever, malaise and severe lymphadenopathy of the neck, inguinal and axillary regions [2–4]. The more virulent strains of MPXV (from the Congo Basin region) can induce mortality rates of ~10% [5–8]. The main clinical difference between human MPX and smallpox is the presentation of lymphadenopathy in the former [9,10]. MPXV is endemic to Central and West Africa with increasing numbers of human infections being reported. In 2003, the transmissibility of MPXV became acutely obvious when an outbreak occurred in the United States and MPXV was transmitted to humans from prairie dogs [11,12]. The global vaccination campaign that eradicated smallpox utilized vaccines that are also efficacious against MPXV and was successful because there is no animal reservoir for variola [13]. Indeed, prior to the eradication of smallpox, the existence of MPXV was unknown and it is likely that many cases of human MPX were reported as smallpox. Cases of human MPX are increasing in Africa, this may be due to several factors: 1) increasing interactions between humans and infected animals through environmental degradation; 2) the cessation of routine vaccination against smallpox; 3) an increase in human susceptibility to the virus; 4) an increase in animal-human and human-human transmissibility of the virus; and 5) adaption of the virus to new host species that co-exist in, or close to, human geographical regions [2,14–18]. The high mortality rate and morbidity rate of MPXV in humans makes it an important emerging disease for study. To date, no antiviral has been FDA-approved for the treatment of human MPX. Vaccines against smallpox have demonstrated good protection against MPXV in animal models and anecdotally in humans [1]; however, most first- and second-generation smallpox vaccines are associated with at least some level of morbidity and a large proportion of the population are contraindicated to vaccination [19]. Third-generation vaccines, such as MVA, have been shown to be safer in non-human primates but require 2 administrations for complete protection [19–22]. Ergo, new and efficacious vaccines to protect against human MPX would be useful.

Using modern genetic engineering techniques, it is possible to isolate, identify, and sequence important genes of pathogenic organisms and place them into new viral vaccine vectors in which they can be faithfully expressed and presented to the immune system with minimal chance of reversion to virulence. In such vaccines, the beneficial properties of both live and killed vaccines can be retained. Viral vectors are not passive delivery systems, but rather active stimulators of the innate and adaptive immunity. Because viral vectors are not all identical, even within the same class of virus, it is difficult to predict from the current knowledge of various potential viral agents which virus will best fulfill the vaccine-vector goals. Consequently, new vaccine-vector agents based on viruses must be explored.

Bovine herpesvirus 4 (BoHV-4) is a gammaherpesvirus no directly associated to a specific disease and establish a persistent infection in the monocyte/macrophage cell lineage of the natural host, the bovine [23]. In contrast to other viruses belonging to the BoHV-4 family, no proof of growth-transformation has been identified and many BoHV-4 genome genes are dispensable and can be potentially deleted to accommodate up to 20–30 kb of foreign DNA [24]. This BoHV-4 characteristic is very important for exploiting BoHV-4 as a vaccine vector.

BoHV-4 have been isolated from other ruminant species such as zebu (*Bos indicus*), American bison (*Bison bison*), African buffalo (*Syncerus caffer*), and sheep. Sporadic isolations were reported in the lion and cat. Experimentally, BoHV-4 was also shown to infect goats, guinea pigs, mice, rats and rabbits. *In vitro*, BoHV-4 is able to replicate in primary cell cultures or cell lines from various animal species such as cattle, sheep, goats, swine, cats, dogs, rabbits, minks, horses, turkeys, ferrets, chickens, hamsters, rats, mice, and monkeys [25–31].

Recombinant BoHV-4s cloned as bacterial artificial chromosome (BAC), expressing immune-dominant antigens from different pathogens, have been shown to successfully immunize mice [32], rats [33], rabbits [29], sheep [27], swine [30] and goats [34]. Moreover, herpes Simplex virus 1 thymidine kinase (HSV-1-TK) gene armed BoHV-4-based vector displayed enhanced oncolytic properties in immune-competent orthotopic syngenic mouse and rats glioma models [31]. We have used the 129 STAT1^(-/-)^ mouse model to evaluate the capability of BoHV-4-based vectors expressing MPXV antigens to elicit a protective immune response against a lethal MPXV challenge [35] [35]. The capacity of the BoHV-4-based vectors to elicit a protective immune response was compared to a smallpox vaccine (MVA) known to induce little to no morbidity [36]. Thus, we have established a starting point for BoHV-4-based vector applications toward MPX and other important human diseases.

## Materials and Methods

### Cell lines

African green Monkey kidney [(VERO), ATCC: CCL-81], bovine embryo kidney [(BEK) from Dr. M. Ferrari, Istituto Zooprofilattico Sperimentale, Brescia, Italy; (BS CL-94)], BEK expressing cre recombinase (BEK*cre*) [28] and Human Embryo Kidney 293T [(HEK 293T) ATCC: CRL-11268] cell lines were cultured in complete growth medium [Eagle's minimal essential medium (EMEM, Lonza) containing 10% fetal bovine serum (FBS), 2 mM of l-glutamine (SIGMA), 100 IU/mL of penicillin (SIGMA), 100 μg/mL of streptomycin (SIGMA) and 2.5 μg/mL of Amphotericin B (SIGMA)] and incubated at 37°C, 5% CO2. *Cercopithecus aethiops* epithelial kidney cells [BS-C-1, (ATCC CCL 26)] were grown in DMEM (Lonza, Basel, Switzerland) containing 10% fetal calf serum (FCS) (Hyclone III, Logan, UT), 2 mM L-glutamine (GIBCO, Grand Island, NY), 100 U/ml penicillin (GIBCO, Grand Island, NY), and 100 μg/ml streptomycin (GIBCO, Grand Island, NY).

### Constructs generation

The A29L, M1R and B6R ORFs, were amplified from Monkeypox DNA (strain V79-I-005; Accession: HQ857562.1) using primer pairs listed in Table 1. The sense primer contains at its 5′-end the *Nhe*I restriction site along with the Kozak’s sequence preceding the ATG, for a better translation initiation, whereas the antisense primer contains at its 5′-end a *Sal*I restriction site for the in-frame cloning of the ORF with a gD106 tag peptide present in pCMV-IgK-VP2-gD_106_ plasmid vector. pCMV-IgK-VP2-gD_106_ has the human cytomegalovirus Immediate Early gene (CMV) promoter, the Immunoglobulin kappa light chain leader sequence (IgK), the Bluetongue virus VP2 ORF, the gD_106_ epitope of Bovine herpesvirus 1 glycoprotein D, successfully used as a tag during the cloning, and the bovine growth hormone polyadenylation site [37].

**Table 1:** List of primers used in that work.

Primer name	5’-3’ sequence	amplicon
A29L sense	5’- ACC*GCTAGC*CCACCATGGACGGAACTCTTTTCCC-3’ NheI	353 bp
A29L antisense	5’-GTA*GTCGAC*CTCATATGGACGCCGTCCAG-3’ SalI	
M1R sense	5’- ACC*GCTAGC*CCACCATGGGTGCCGCAGCAAGCAT-3’ NheI	773 bp
M1R antisense	5’-GTA*GTCGAC*GTTTTGTATATCCGTGGTAG-3’ SalI	
B6R sense	5’- ACC*GCTAGC*CCACCATGAAAACGATTTCCGTTGT-3’ NheI	977 bp
B6R antisense	5’-GTA*GTCGAC*CGGTAGCAATTTATGGAACT-3’ SalI	
EF1α-NdeI-sense	5’-CCC*CATATG*CTTGAAAGGAGTGGGAATTGG-3’ NdeI	1237 bp
EF1α-NheI-antisense	5’-CCC*GCTAGC*TAAATTTAAATGTCGAAATTCC-3’ NheI	

The 3 ORFs were cut with *Nhe*I/*Sal*I and cloned into pCMV-IgK-VP2-gD_106_ cut with the same enzymes. This strategy allowed the substitution of the IgK-VP2 sequence with those of the 3 ORFs to generate pCMV-A29LgD_106_, pCMV-M1RgD_106_ and pCMV-B6RgD_106_.

To generate pTK-CMV-A29LgD_106_-TK, pCMV-A29LgD_106_ was cut with *Bam*HI, treated with T4 DNA polymerase for blunt ending and cut with *Nhe*I; A29LgD_106_ (442 bp) was excised from the pCMV-A29LgD_106_ and inserted into the shuttle vector pINT2EGFP [25] cut with *Nhe*I/*Sma*I to replace GFP ORF with the chimeric protein. pINT2EGFP contains two BoHV-4 Thymidine kinase gene homology regions flanking the Green Fluorescent Protein (GFP) expression cassette driven by the CMV promoter, cutting with *Nhe*I/*Sma*I the GFP ORF was replaced with that of A29LgD_106_.

To generate pEF1α-M1RgD_106_ and pEF1α-B6RgD_106_, the CMV promoter of pCMV-M1RgD_106_ and pCMV-B6RgD_106_ was substituted with that of the human elongation factor 1 alpha (EF1α). EF1α was amplified by PCR from pWPI plasmid vector (Addgene plasmid #12254). pWPI was first linearized with *Pme*I, then the PCR reaction was carried out with 0.25 μM of a couple of primers (EF1α-NdeI-sense and EF1α-NheI-antisense; Table 1) in a final volume of 50 μl containing 10 mM Tris—hydrochloride pH 8.3, 0.2 mM deoxynucleotide triphosphates, 3 mM MgCl2, 50 mM KCl and 5% DMSO. Each cycle of 35, consisted of denaturation at 94°C for 1 min, primer annealing at 50°C for 1 min and elongation for 90 sec with 1U of Pfu DNA polymerase at 72°C. The 1237 bp EF1α amplification product was checked in 1% agarose gel and visualized after ethidium bromide staining in 1× TAE buffer (40 mM Tris-acetate, 1 mM EDTA).

pTK-EF1α-M1RgD_106_-TK and pTK-EF1α-B6RgD_106_-TK constructs were obtained by sub-cloning EF1α-M1RgD_106_ and EF1α-B6RgD_106_ expression cassettes, cut with *Nde*I/*Mlu*I and blunted-end with T4 DNA polymerase, from pEF1α/M1RgD_106_ and EF1α/B6RgD_106_ respectively in *Sma*I cut pINT2 [38]. All enzymes were purchased from Thermo Scientific.

### Transient transfection

Confluent HEK293T cells were seeded into 6 well plates (3x10^5^ cells/well) and incubated at 37°C with 5% CO_2_; when the cells were sub-confluent the culture medium was removed and the cells were transfected with pTK-CMV-A29LgD_106_-TK, pTK-EF1α-M1RgD_106_-TK or pTK-EF1α-B6RgD_106_-TK using Polyethylenimine (PEI) transfection reagent (Polysciences, Inc.). Briefly, 3 μg of DNA were mixed with 7.5 μg PEI (1mg/mL) (ratio 1:2.5 DNA- Pei) in 200 μL of Dulbecco’s modified essential medium (DMEM) at high glucose percentage (Euroclone) without serum. After 15 min at RT, 800 μL of medium without serum was added and the transfection solution was transferred to the well and left on the cells for 6 h at 37°C with 5% CO_2_ in air, in a humidified incubator. The transfection mixture was then replaced with fresh medium (EMEM, with 10% FBS, 50 IU/ml of penicillin, 50 μg/ml of streptomycin and 2.5 μg/ml of Amphotericin B) and incubated for 24 h at 37°C with 5% CO_2_.

### Viruses and viral replication

BoHV-4-A, BoHV-4-A-CMV-A29LgD_106_ΔTK, BoHV-4-A-EF1α-M1RgD_106_ΔTK and BoHV-4-A-EF1α-B6RgD_106_ΔTK were propagated by infecting confluent monolayers of BEK and VERO cells at a multiplicity of infection (MOI) of 0.5 50% tissue culture infectious doses (TCID_50_) per cell and maintained in medium with only 2% FBS for 2 h. The medium was then removed and replaced with fresh EMEM containing 10% FBS. When the cytopathic effect (CPE) occurred in the majority of the cell monolayer (∼72 h post infection), the virus was prepared by freezing and thawing cells three times and pelleting the virions through a 30% sucrose cushion, as described previously [39]. Virus pellets were then resuspended in cold EMEM without FBS. TCID_50_ were determined with BEK cells by limiting dilution. A plaque-purified isolate of the MPXV strain ZAI-79 [40] was purified through a sucrose cushion [41] and propagated in BS-C-1 cells [42]. Virus infectivity was estimated as described previously [43]. Briefly, virus suspensions were serially diluted in PBS +1% FCS (Fetal Clone II, HyClone), absorbed to monolayers for 1 hour at 37°C, and overlaid with a suspension of 1% carboxyl methyl cellulose in DMEM +5% FCS. After 2 days at 37°C, virus plaques were visualized and virus inactivated by the addition to each well of a 0.3% crystal violet/10% formalin solution.

### Western immunoblotting

Protein cell extracts were obtained from a 6-well confluent plate of HEK293T transfected with pINT2/CMVA29LgD_106_, pINT2/EF1αM1RgD_106_ or pINT2/EF1αB6RgD_106_ and from 25-cm^2^ confluent flasks of BEK cells or VERO cells infected with BoHV-4-A-CMV-A29LgD_106_ΔTK, BoHV-4-A-EF1α-M1RgD_106_ΔTK and BoHV-4-A-EF1α-B6RgD_106_ΔTK by adding 100 μL of cell extraction buffer (50 mM Tris—HCl, 150 mM NaCl, and 1% NP-40; pH 8). A 10% SDS—PAGE gel electrophoresis was used to analyze cell extracts containing 50 μg of total protein, after protein transfer in nylon membranes by electroblotting, the membranes were incubated with bovine anti gD_106_ monoclonal antibody (clone 1B8-F11; VRMD, Inc., Pullman, WA) antibody, probed with horseradish peroxidase-labelled anti-mouse immunoglobulin antibody (Sigma) and visualized by enhanced chemiluminescence (ECL Kit; Pierce).

### BAC recombineering and selection

Recombineering was performed as previously described [44] with some modifications. Five hundred microliters of a 32°C overnight culture of SW102 containing BAC-BoHV-4-A-Kana-GalKΔTK, were diluted in 25 ml Luria—Bertani (LB) medium with or without chloramphenicol (SIGMA) selection (12.5 mg/ml) in a 50 ml baffled conical flask and grown at 32°C in a shaking water bath to an OD_600_ of 0.6. Then, 10 ml were transferred to another baffled 50 mL conical flask and heat-shocked at 42°C for exactly 15 min in a shaking water bath. The remaining culture was left at 32°C as the uninduced control. After 15 min the two samples, induced and uninduced, were briefly cooled in ice/water bath slurry and then transferred to two 15mL Falcon tubes and pelleted using 5000 r.p.m. (eppendorf centrifuge) at 0°C for 5 min. The supernatant was poured off and the pellet was resuspended in 1mL ice-cold ddH_2_O by gently swirling the tubes in ice/water bath slurry. Subsequently, 9 ml ice-cold ddH_2_O were added and the samples pelleted again. This step was repeated once more, the supernatant was removed and the pellet (50 μL each) was kept on ice until electroporated with gel-purified fragments (∼3.3, ∼4.4 and ∼4.6 kb respectively for TK-CMV-A29LgD_106_-TK, TK-EF1α-M1RgD_106_-TK and TK-EF1α-B6RgD_106_-TK) obtained by cutting pTK-CMV-A29LgD_106_-TK, pTK-EF1α-M1RgD_106_-TK and pTK-EF1α-B6RgD_106_-TK with *Cla*I/*Pvu*II (Thermo Scientific). An aliquot of 25 μl (~200 ng) was used for each electroporation in a 0.1 cm cuvette at 25 μF, 2.5 kV and 201Ω. After electroporation, for the counter selection step, the bacteria were recovered in 10 mL LB in a 50 mL baffled conical flask and incubated for 4.5h in a 32°C shaking water bath. Bacteria serial dilutions were plated on M63 minimal medium plates containing 15 g/L agar, 0.2% glycerol, 1mg/L D-biotin, 45 mg/L L-leucine, 0.2% 2- deoxy-galactose and 12.5 mg/mL chloramphenicol. All the complements for M63 medium were purchased from SIGMA.

Plates were incubated 3–5 days at 32°C; then several selected colonies were picked up, streaked on McConkey agar indicator plates (DIFCO, BD Biosciences) containing 12.5 g/mL of chloramphenicol and incubated at 32°C for 3 days until white colonies appeared. White colonies were grown in duplicate for 5–8h in 1mL of LB containing 50 mg/mL of kanamycin (SIGMA) or LB containing 12.5 mg/mL of chloramphenicol. Only those colonies that were kanamycin negative and chloramphenicol positive were kept and grown overnight in 5mL of LB containing 12.5 mg/mL of chloramphenicol. BAC DNA was purified and analyzed through *Hind*III restriction enzyme digestion for TK-CMV-A29LgD_106_-TK, TK-EF1α-M1RgD_106_-TK and TK-EF1α-B6RgD_106_-TK fragment targeted integration, was separated by electrophoresis overnight in a 1% agarose gel, stained with ethidium bromide, capillary transferred to a positively charged nylon membrane (Roche), and cross-linked by UV irradiation by standard procedures [28]. Hybridization with digoxigenin-labeled probes confirmed the identity of specific restriction fragments.

The 353, 573, 977 bp amplicons for A29L, M1R and B6R probes were generated by PCR with the primers: A29L sense/antisense, M1R sense/antisense, and B6R sense/antisense listed in Table 1, as previously described [29]. Original detailed protocols for recombineering can also be found at the recombineering website (http://recombineering.ncifcrf.gov).

### Cell culture electroporation and recombinant virus reconstitution

BEK or BEKcre cells were maintained as a monolayer with complete DMEM growth medium with 10% FBS, 2 mM L-glutamine, 100 IU/mL penicillin and 10 mg/mL streptomycin. When cells were sub-confluent (70–90%) they were split to a fresh culture vessel (i.e., every 3–5 days) and were incubated at 37°C in a humidified atmosphere of 95% air–5% CO2. BAC DNA (5 μg) was electroporated in 600 μl DMEM without serum (Equibio apparatus, 270 V, 960 mF, 4-mm gap cuvettes) into BEK and BEKcre cells from a confluent 25-cm^2^ flask. Electroporated cells were returned to the flask, after 24h the medium was replaced with fresh medium, and cells were split 1:2 when they reached confluence at 2 days post-electroporation. Cells were left to grow until the appearance of CPE. Recombinant viruses were propagated by infecting confluent monolayers of BEK cells at a M.O.I. of 0.1–0.5 TCID_50_ per cell and maintaining them in MEM with 10% FBS for 2 h.

### Viral growth curves

BEK cells were infected with BoHV-4-A, BoHV-4-A-CMV-A29LgD_106_ΔTK, BoHV-4-A-EF1α-M1RgD_106_ΔTK and BoHV-4-A-EF1α-B6RgD_106_ΔTK at a M.O.I. of 0.1 TCID50/cell and incubated at 37°C for 4 h. Infected cells were washed with serum-free EMEM and then overlaid with EMEM containing 10% FBS, 2 mM Lglutamine, 100 IU/mL penicillin, 100 mg/mL streptomycin and 2.5-mg/mL Amphotericin B. The supernatants of infected cultures were harvested after 24, 48, 72 and 96 h, and the amount of infectious virus was determined by limiting dilution on BEK cells by the TCID50 method.

### Animals

Eight-week old female 129 *stat1* ^-/-^ mice were bred in-house and housed in filter-top microisolator cages and fed commercial mouse chow and water *ad libitum*. The randomized mice were housed in an animal biosafety level 3 containment area, with 5 mice/group. Animal husbandry and experimental procedures were approved by the Institutional Animal Care and Use Committee. Mice were monitored every day until the termination of the experiment.

### Ethics statement

Animal Use Protocol 2082 entitled “Pathogenesis of orthopoxviruses and efficacy testing of orthopoxvirus vaccines and antivirals " was approved by the Saint Louis University Institutional Animal Care and Use Committee (IACUC) on 5/24/2012. Saint Louis University is a USDA registered research facility (43-R-011), is regularly inspected and files all required documentation, including an annual report. In addition, under the provisions of the Public Health Service Policy on the Humane Care and Use of Laboratory Animals, the University files required assurance documents to the Office of Laboratory Animal Welfare (OLAW). (OLAW Assurance Number A-3225-01, effective from March 4, 2013 through March 31, 2017). The Animal Care and Use Program at Saint Louis University are fully accredited by the Association for Assessment and Accreditation of Laboratory Animal Care, International (AAALACI), continuing accreditation notification July 18, 2012.

### Vaccines and vectors

BoHV-4s vectors were injected intraperitoneally (IP) in a total volume of 300 μl with DMEM used as a vehicle. For vaccinations with one vector, injections were comprised of 100 #x03BC;l of vector + 200 μl of vehicle. For the combination injections, 100 #x03BC;l of each vector was included for a total of 300 #x03BC;l Injections were given as a primary vaccination at T = 0 days and as a booster vaccination at T = 23 days (see Table 2). Each vector was injected at a Modified Vaccinia Ankara (MVA) (a gift from the NIAID-NIH, Bethesda, MD) was provided at a dose of 2x10^8^ plaques forming units (PFU)/ml and was injected in 0.1 ml between the skin and underlying layers of tissue in the scapular region on the backs of mice.

**Table 2:** Study design and mortality of mice challenged via the IN route with 2x10^5^ PFU of MPXV.

Group	Cage	No. of Mice	Vaccine (T = 0)^a^	Booster (T = 23)^b^	MPXV Challenge (T = 50)^c^	Day of Death (RTC)^d^	MTD±SEM^d^	Mortality (%)
1	1	5	N/T	N/T	PBS			
2	2	5	N/T	N/T	+	7,8,8,9,9	8.2±0.4	100
3	3	5	Veh	Veh	+	7,9,9,9,17	10.2±1.7	100
4	4	5	MVA	Veh				
	5	5	MVA	MVA	+			
5	6	5	A29L	Veh		8,9,9,9	8.8±0.3	80
	7	41	A29L	A29L	+	7,10,10,	9.0±1.0	75
6	8	5	M1R	Veh		7,8	7.5±0.5	40
	9	5	M1R	M1R	+			
7	10	5	B6R	Veh		6,8,9	7.7±0.9	60
	11	4^e^	B6R	B6R	+	7,8	7.5±0.5	50
8	12	5	Combo	Veh		7,8	7.5±0.5	40
	13	5	Combo	Combo	+	9	9.0	20

^a^BoHV-4 vaccines were administered at T = 0 days via the IP route in a total volume of 0.3 ml at 1x10^7^ PFU. MVA was administered at a dose of 2x10^7^ PFU in a total volume of 0.1 ml injected IM. Veh indicates a DMEM vehicle.

^b^Booster vaccinations were administered as for ^a^.

^c^Mice were challenged via the IN route with 2x10^5^ PFU of MPXV. Virus was introduced in a total volume of 25 #x03BC;l to one nare.

^d^RTC, relative to challenge; MTD, mean time to death; SEM, standard error of the mean.

^e^One mouse was removed due to mortality during blood collection.

### MPXV challenge

Mice were anesthetized with 0.1 ml/10 g body weight of ketamine HCl (6 mg/ml) and xylazine (0.5 mg/ml) by intraperitoneal injections. Anesthetized mice were laid on their dorsal side with their bodies angled so that the anterior end was raised 45° from the surface; a plastic mouse holder was used to ensure conformity [45]. MPXV was diluted in PBS without Ca^2+^ and Mg^2+^ to the required concentration and slowly loaded into each nare (5 #x03BC;l/nare). Mice were subsequently left in situ for 2–3 minutes before being returned to their cages.

### Statistics

Paired T-tests (tailed) were used to compare means between groups of mice. Mortality rates were compared using the Fisher’s exact test. Blinded lesion pictures were measured qualitatively using a scoring system ranging 0–4 in severity. P values <0.05 were considered statistically significant.

## Results

### Design and expression of monkeypox virus tagged peptides

**Fig 1:**
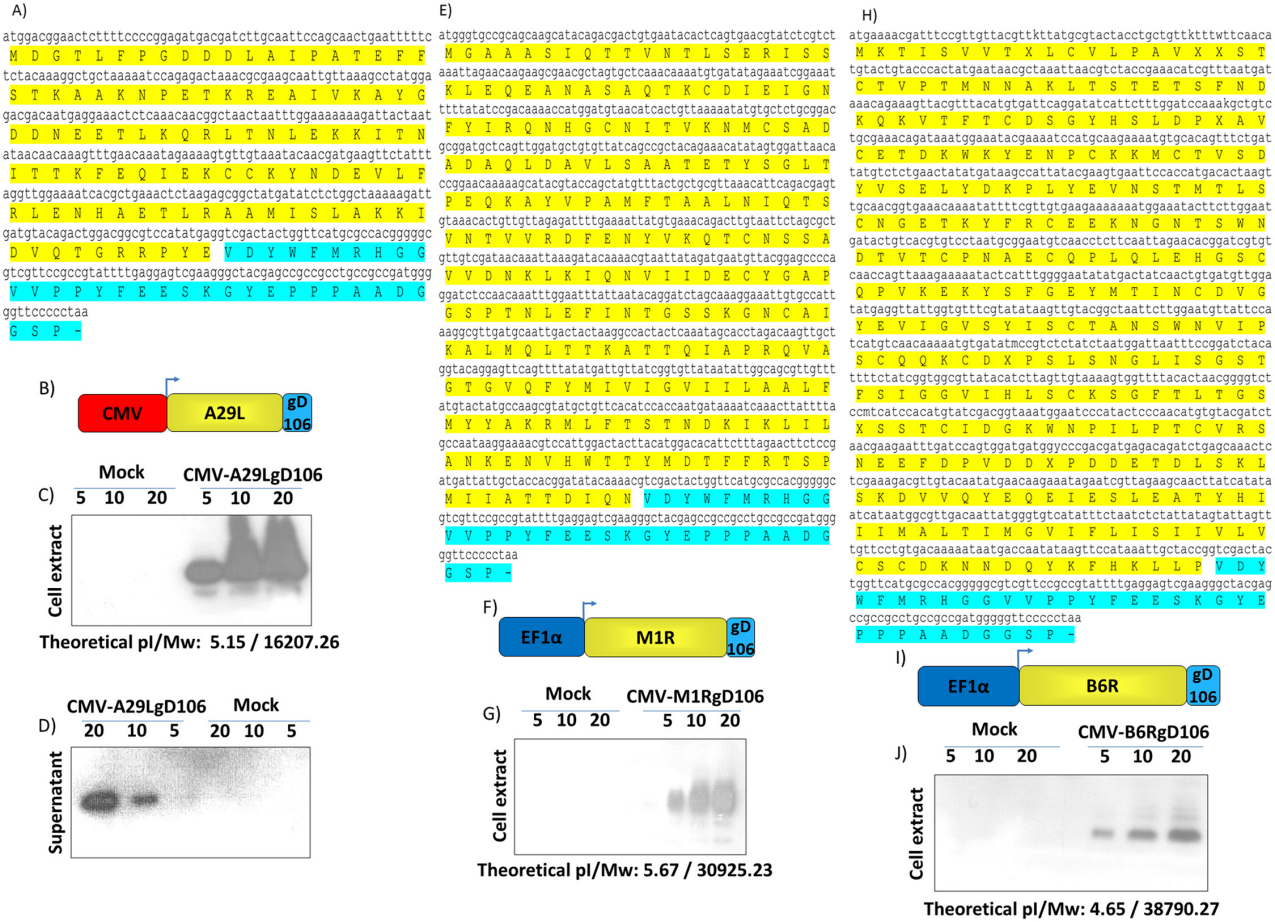
Design and expression of MPXV glycoproteins. A29L **(A)**, M1R **(E)** and B6R **(H)** gD_106_ peptide tagged ORFs full length sequence with the deduced amino acid composition (yellow and baby blue for gD_106_), along with the diagram (not on scale) of their expression cassette **(B, F and I)** driven by CMV (red) or EF1α (blue) promoter. Western immunoblotting of cells, transfected with CMV-A29LgD_106_ **(C)**, EF1α-M1RD_106_ **(G)** and EF1α-B6RD_106_ **(J)**. The lanes were loaded with different amounts of total protein cell extract (5, 10 and 20 μg). Negative control was established with pEGFP-1 transfected cells (Mock). **D)** Western immunoblotting of CMV-A29LgD_106_ transfected cells, in this case the lanes were loaded with different amounts (5, 10 and 20 μl) of serum free cells supernatant. As before, negative control was established with pEGFP-1 transfected cells (Mock) supernatant. EF1α-M1RD_106_ and EF1α-B6RD_106_ transfected cells supernatant was tested too, but not shown because the transgenic product was undetectable and thus not secreted.

Among the approximately 200 genes that comprise the Monkeypox virus genome, only few genes encoding antigenic proteins—that are known—are able to elicit a neutralizing antibody response in vaccinated animals. Among these antigens, A29L, M1R and B6R orthologs were selected as candidate antigens to be delivered by BoHV-4 based-vector. A29L, M1R and B6R ORFs were amplified by PCR from a Cosmid library and sub-cloned in frame with a tag peptide, gD106 [46] (Fig 1A, 1E and 1H), which was contained in a mammalian expression vector plasmid construct. M1R and B6RgD_106_ tagged ORFs were placed under the transcriptional control of the EF1α promoter (Fig 1F and 1I), whereas A29LgD_106_ tagged ORF under the transcriptional control of the CMV promoter (Fig 1B). So generated expression cassettes (CMV-A29LgD_106_, EF1α-M1RgD_106_ and EF1α-B6RgD_106_) were validated, in terms of protein expression, by transient transfection in 293T cells and Western-immunoblotting with a monoclonal antibody directed against the gD_106_ tag peptide. A29L, M1R and B6RgD_106_ tagged antigens were all well expressed in the whole cell extracts of the transiently transfected cells (Fig 1C, 1G and 1J), further A29LgD_106_ was also secreted (Fig 1D) in the supernatant of the transiently transfected cells.

### Construction of BoHV-4s-based vector expressing A29L, M1R and B6RgD106 tagged antigens

**Fig 2:**
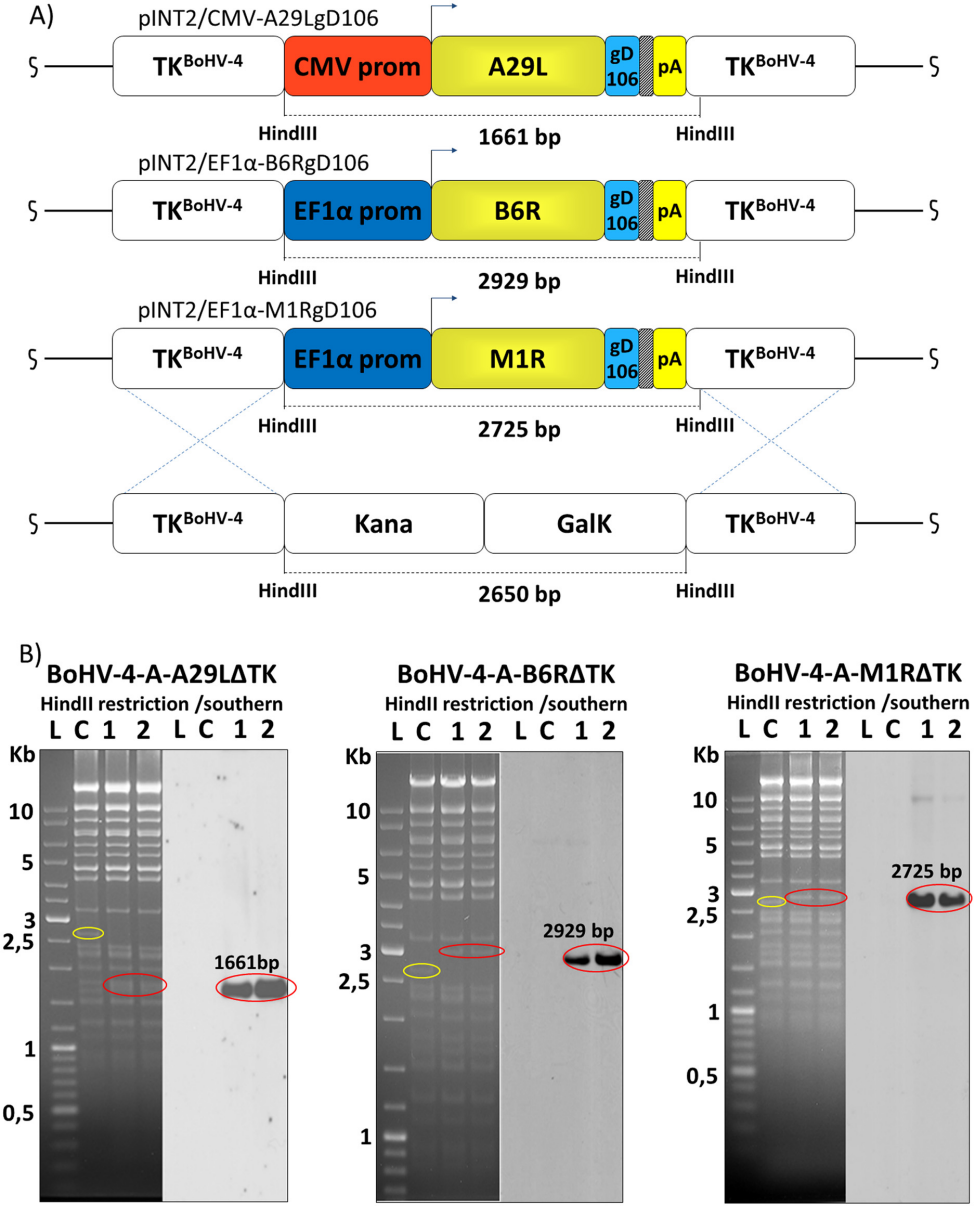
Generation of recombinant BoHV-4s. Diagram (not to scale; **A**) showing the retargeting event obtained by heat-inducible homologous recombination in SW102 containing pBAC-BoHV-4-A-TK-KanaGalK-TK, where the Kana/GalK cassette was replaced with the CMV-A29LgD_106_, EF1α-M1RgD_106_ and EF1α-B6RgD_106_ expression cassettes flanked by BoHV-4 TK sequences, located in pINT2 shuttle plasmid vector. **B**) Representative 2-deoxy-galactose resistant colonies (1 and 2) tested by HindIII restriction enzyme analysis, agar gel electrophoresis, and Southern blotting performed with specific probes for the A29L, M1R and B6R ORFs. The 2,650 bp band (yellow circle), corresponding to the un-retargeted pBAC-BoHV-4-A-TK-KanaGalK-TK control (C), has been replaced by a 1,661 bpband (red circle) in pBAC-BoHV-4-A-CMV-A29LgD_106_ΔTK, by a 2,929 bp band (red circle) in pBAC-BoHV-4-A-EF1α-B6RgD_106_ΔTK and by a 2,725 bp band (red circle) in pBAC-BoHV-4-A-EF1α-M1RgD_106_ΔTK.

An apathogenic strain of BoHV-4 cloned as a BAC was used to create a BoHV-4-A-based vector [28]. The TK BoHV-4-A genome locus was utilized as the integration site for the CMV-A29LgD_106_, EF1α-M1RgD_106_ and EF1α-B6RgD_106_ expression cassettes. The BoHV-4 TK genomic region is strongly conserved among BoHV-4 isolates [47], ensuring the stability of the genomic locus from potential recombination when foreign DNA sequences are inserted in. Indeed, the BoHV-4 TK genomic locus has been interrupted by the insertion of foreign DNA sequences of different size, without interfering with viral replication *in vitro* and loss of heterologous protein expression [25,27–29,31,32,34]. CMV-A29LgD_106_, EF1α-M1RgD_106_ and EF1α-B6RgD_106_ expression cassettes were first sub-cloned into pINT2, a shuttle vector plasmid containing 2 BoHV-4 TK sequences [25], to generate pTK-CMV-A29LgD_106_-TK, pTK-EF1α-M1RgD_106_-TK and pTK-EF1α-B6RgD_106_-TK targeting vectors. Restriction enzyme linearized targeting vectors were used for heat-inducible homologous recombination SW102 *E*. *Coli* containing pBAC-BoHV-4-A-KanaGalKΔTK [28,39,48] (Fig 2A) to generate pBAC-BoHV-4-A-CMV-A29LgD_106_ΔTK, pBAC-BoHV-4-A-EF1α-M1RgD_106_ΔTK and pBAC-BoHV-4-A-EF1α-B6RgD_106_ΔTK. Selected clones were first analyzed by *Hind*III restriction enzyme digestion and then by southern blotting (Fig 2B). Because heat-inducible recombination in SW102 *E*. *Coli* and repeated passages could establish altered bacterial phenotypes due to an aberrant recombenases transcription, SW102 *E*. *Coli* carrying pBAC-BoHV-4-A-CMV-A29LgD_106_ΔTK, pBAC-BoHV-4-A-EF1α-M1RgD_106_ΔTK and pBAC-BoHV-4-A-EF1α-B6RgD_106_ΔTK were serially cultured over for 20 passages and checked by *Hind*III restriction enzyme digestion. No differences among restriction patterns at various passages were detected (S1 Fig), thus ensuring the stability of the clones.

### Recombinant BoHV-4s reconstitution and characterization

**Fig 3:**
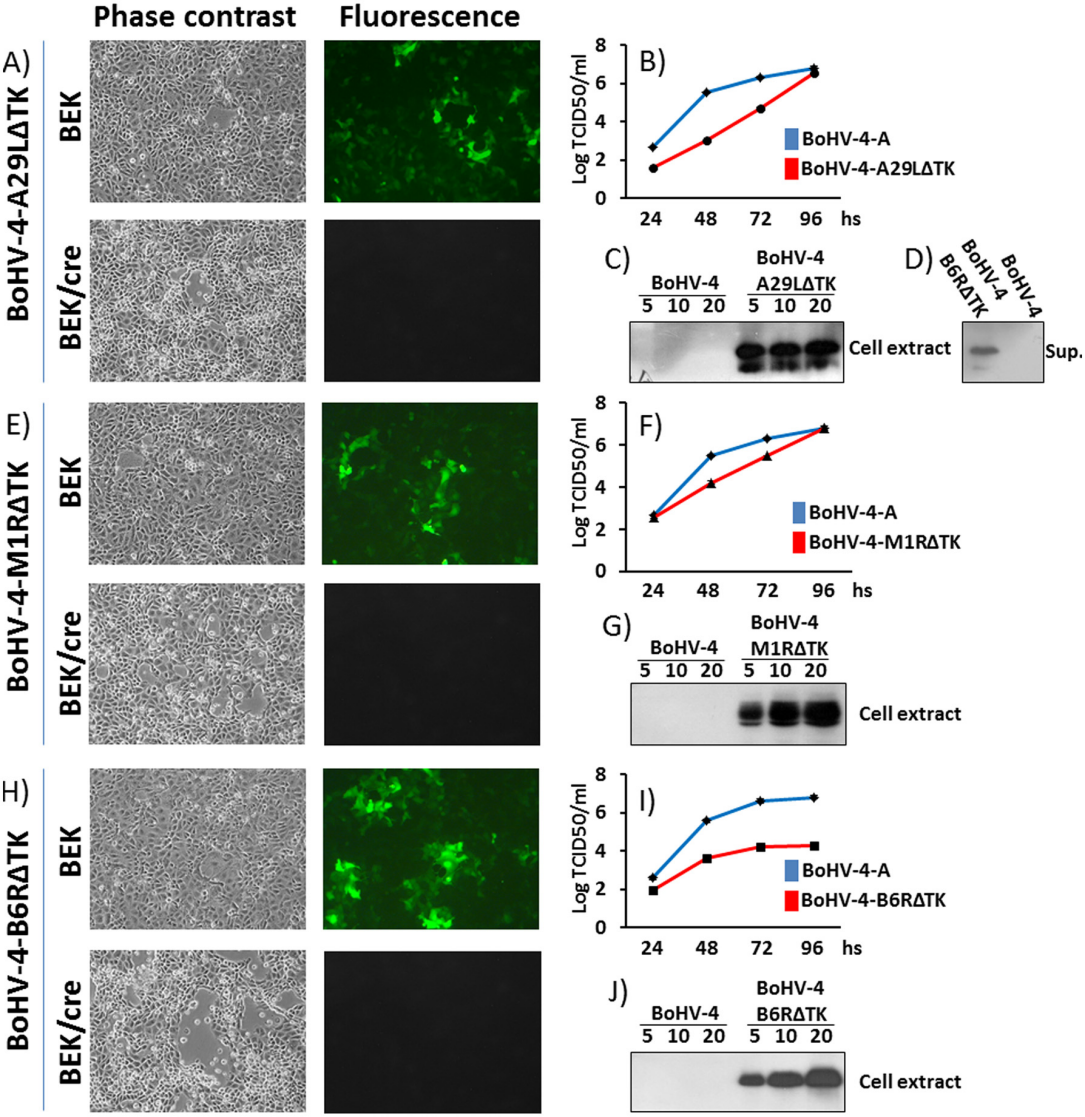
Reconstitution and characterization of recombinant viruses. Representative phase contrast and fluorescent microscopic images of plaques formed by viable reconstituted recombinant BoHV-4-A-CMV-A29LgD_106_ΔTK **(A)**, BoHV-4-A-EF1α-M1RgD_106_ΔTK **(E)** and BoHV-4-A-EF1α-B6RgD_106_ΔTK **(H)** after the corresponding BAC DNA electroporation into BEK cells or in BEK cells expressing cre recombinase (Magnification, ×10). Replication kinetics of BoHV-4-A-CMV-A29LgD_106_ΔTK **(B)**, BoHV-4-A-EF1α-M1RgD_106_ΔTK **(F)** and BoHV-4-A-EF1α-B6RgD_106_ΔTK **(I)** growth on BEK cells, compared with those of the parental BoHV-4-A isolate. The data presented are the means ± standard errors of triplicate measurements (*P*>0.05 for all time points as measured by Student's *t* test). Western immunoblotting of cells, infected with BoHV-4-A-CMV-A29LgD_106_ΔTK **(C)**, BoHV-4-A-EF1α-M1RgD_106_ΔTK **(G)** and BoHV-4-A-EF1α-B6RgD_106_ΔTK **(J)**. The lanes were loaded with different amounts of total protein cell extract (5, 10 and 20 μg). Negative control was established with BoHV-4-A infected cells. **D)** Western immunoblotting of BoHV-4-A-CMV-A29LgD_106_ΔTK infected cells; in this case the lanes were loaded with different amounts (5, 10 and 20 μl) of serum free cells supernatant. As before, negative control was established with BoHV-4-A infected cells supernatant. BoHV-4-A-EF1α-M1RgD_106_ΔTK and BoHV-4-A-EF1α-B6RgD_106_ΔTK infected cells supernatant were tested too, but not shown because the transgenic product was undetectable and thus not secreted.

Infectious viable BoHV-4-A-CMV-A29LgD_106_ΔTK, BoHV-4-A-EF1α-M1RgD_106_ΔTK and BoHV-4-A-EF1α-B6RgD_106_ΔTK were obtained by electroporating pBAC-BoHV-4-A-CMV-A29LgD_106_ΔTK, pBAC-BoHV-4-A-EF1α-M1RgD_106_ΔTK and pBAC-BoHV-4-A-EF1α-B6RgD_106_ΔTK DNA into BEK and BEK*cre* cells. The only difference was that the recombinant viruses reconstituted from electroporated BEK*cre* resulted in depletion of the BAC plasmid backbone containing the GFP expression cassette, as shown by the loss of GFP expression (Fig 3A, 3E and 3H). Because the time necessary to reconstitute the viable recombinant BoHV-4s was different among them, it was of interest to know if the foreign antigens, encoded by the expression cassette integrated into the viral genome, could have a detrimental effect on the viral replication. Therefore, the growth characteristics of BoHV-4-A-CMV-A29LgD_106_ΔTK, BoHV-4-A-EF1α-M1RgD_106_ΔTK and BoHV-4-A-EF1α-B6RgD_106_ΔTK were compared with that of the parental virus, BoHV-4-A. Although BoHV-4-A-CMV-A29LgD_106_ΔTK and BoHV-4-A-EF1α-M1RgD_106_ΔTK demonstrated a slower replication kinetics respect to BoHV-4-A, they reached the same viral titer at the end-point (~10^6^) (Fig 3B and 3F). Whereas BoHV-4-A-EF1α-B6RgD_106_ΔTK replication was drastically impaired, a 2 log reduction of the viral titer end-point (~10^4^) respect to BoHV-4-A was observed (Fig 3I); however, transgene expression was well detected in the whole cell extract of BoHV-4-A-CMV-A29LgD_106_ΔTK, BoHV-4-A-EF1α-M1RgD_106_ΔTK and BoHV-4-A-EF1α-B6RgD_106_ΔTK infected cells (Fig 3C, 3G and 3J). Further, A29LgD_106_ glycoprotein was found to be expressed as a secreted protein in the supernatant of BoHV-4-A-CMV-A29LgD_106_ΔTK infected cells (Fig 3D).

### *In vivo* efficacy testing of BoHV-4-A-CMV-A29LgD_106_ΔTK, BoHV-4-A-EF1α-M1RgD_106_ΔTK and BoHV-4-A-EF1α-B6RgD_106_ΔTK

To test the efficacy of the vectors *in vivo*, we sought to determine if they could protect mice against a lethal challenge with MPXV. Several murine strains have been developed as models of MPXV, in this study we utilized the 129 *stat1* ^*-/-*^ strain. Thirteen cages were prepared with 5 mice/cage (Table 2). Cages 1 and 2 were un-vaccinated. Cage 3 was vaccinated with vehicle (DMEM without FBS). Cages 4 and 5 were vaccinated with MVA where cage 4 received a primary injection of vaccine at T = 0 days and a vehicle booster at T = 23 days, and cage 5 received a primary injection of vaccine at T = 0 days and a booster injection of vaccine at T = 23 days. Similar vaccination regimens were performed on cages 6 and 7 (vaccinated with BoHV-4-A-CMV-A29LgD_106_ΔTK), cages 8 and 9 (vaccinated with BoHV-4-A-EF1α-M1RgD_106_ΔTK) and cages 10 and 11 (vaccinated with BoHV-4-A-EF1α-B6RgD_106_ΔTK). Cages 12 and 13 were also vaccinated following the above regimen; however, these mice received a combination (combo) of the 3 BoHV-4 vectors. BoHV-4 vectors were injected IP in a total volume of 0.3 ml. There was no apparent morbidity—as measured visually—or weight-loss recorded in any of the mice (S2 Fig).

**Fig 4:**
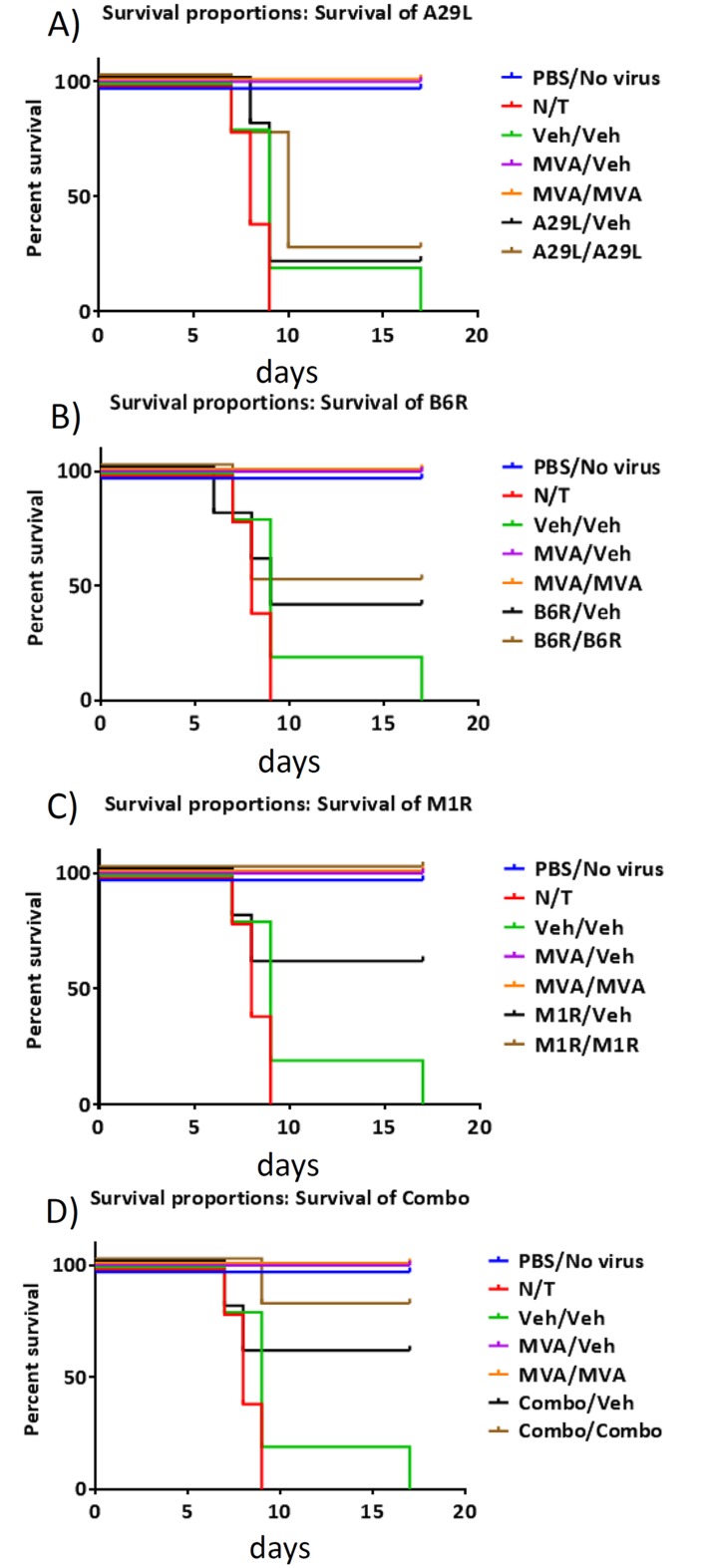
Mortality in MPXV challenged mice. Vaccinated and control mice were challenged with 2x10^5^ PFU of MPXV 50 days following the initial vaccination with MVA or BoHV-4 vectors A29L **(A)**, M1R **(B)**, B6R **(C)**, or a combination of all 3 vectors **(D)**. Mice were monitored for mortality. N = 5 mice per group.

**Fig 5:**
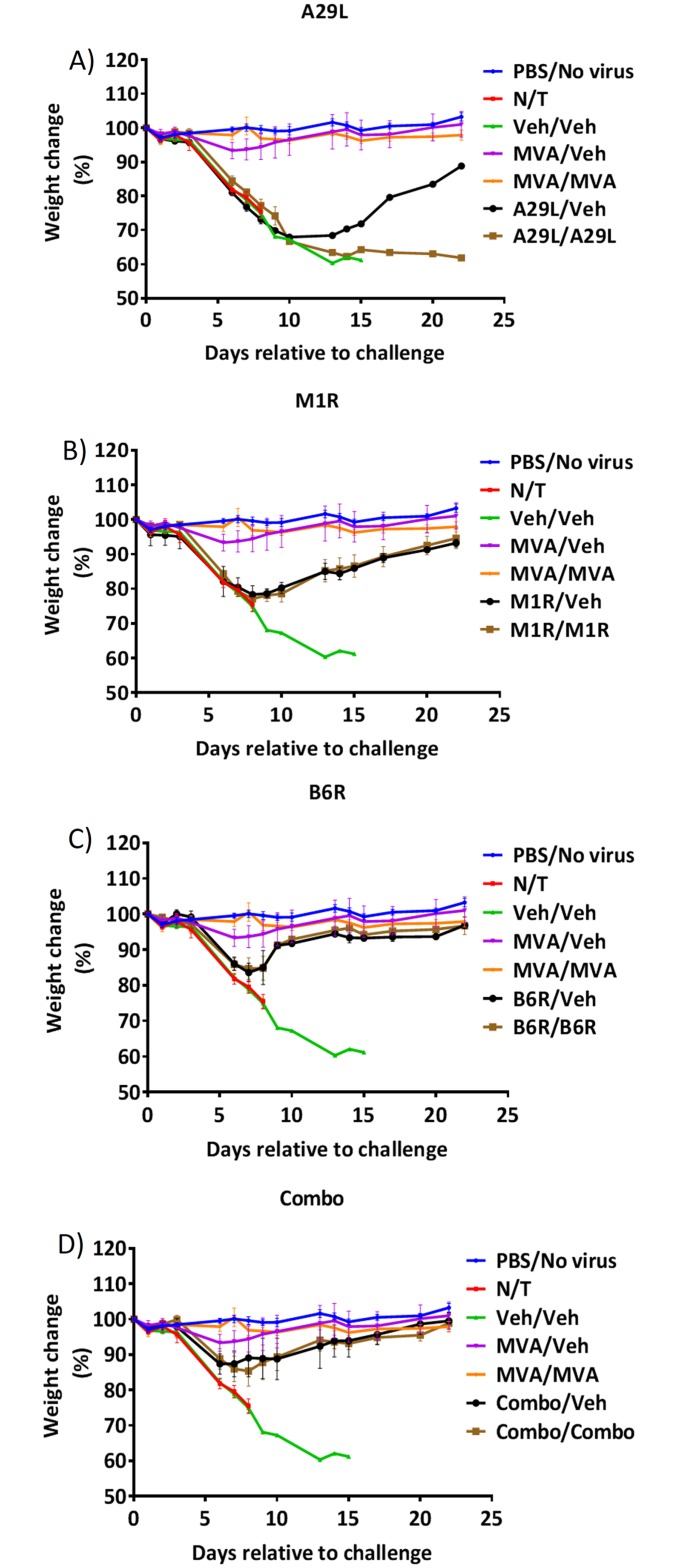
Weight-change in MPXV challenged mice. Vaccinated and control mice were challenged with 2x10^5^ PFU of MPXV 50 days following the initial vaccination with MVA or BoHV-4 vectors A29L **(A)**, M1R **(B)**, B6R **(C)**, or a combination of all 3 vectors **(D)**. Mice were monitored for weight-change. Error bars indicate SEM. N = 5 mice per group.

At T = 50 days, mice in cages 2–13 were challenged with 2x10^5^ PFU/mouse of MPXV. Mortality rates are shown in Fig 4. As expected, the MVA vaccinated mice in cages 4 (MVA/Veh) and 5 (MVA/MVA) were 100% protected against challenge. Mice in cage 9 (M1R/M1R) were also 100% protected (P = 0.004); and although mice in cage 13 (Combo/Combo) experienced 1 death, they were still 80% protected against the MPXV challenge (P = 0.02). When comparing weight-change (Fig 5), we found that mice in cage 5 (MVA/MVA) did not lose weight compared to the PBS control (cage 1) and that mice in cage 4 (MVA/Veh) only lost weight (≤5%) on days 6, 7 and 8 post challenge. We also found that mice in cages 10 (B6R/Veh), 11 (B6R/B6R), 12 (Combo/Veh), and 13 (Combo/Combo) had significantly reduced weight-loss from day 8 post challenge (15%, 15%, 11%, and 15% on day 8, respectively), even though mice in cage 13 were 80% protected against the challenge. Some protection from weight-loss was also afforded to mice in cages 8 (M1R/veh) and 9 (M1R/M1R) on days 13, 14 and 15 post challenge (≤15%).

These data indicate that the presence of M1R is required to protect the mice against challenge, and that a booster vaccination is required. It also indicates that a combination of the 3 vectors improves protection against weight-loss. Although M1R could provide protection, it was inferior to that provided by vaccination with MVA. The data also indicate that although B6R does not provide protection against mortality, it does provide protection against weight-loss in surviving mice (see above).

## Discussion

The aim of the current study was to ascertain the potential utility of Bovine herpesvirus 4 (BoHV-4)-based vectors as safe, potent, large-capacity vaccine vectors for category A agents—MPXV for this specific case. This study demonstrated protection in the STAT1^-/-^ model and consideration should be given to also evaluating the vectors in CAST/EiJ mice which have also been established as a murine model of monkeypox [49,50]. Ultimately, this study could pave the way for further studies in other animal models such as prairie dogs (*Cynomys ludovicianus*) [51–53], the 13-lined ground squirrel (*Spermophilus tridecemlineatus*) [54,55] and non-human primates (reviewed in [1])—all of which have been extensively used as models of human monkeypox—and ultimately in human protection studies. In fact, since MPXV also infects humans and causes clinical-signs very similar to smallpox, it is classified as a category A select agent [56]. Primarily for ethical but also for cost reasons, C57BL/6 STAT1 knockout mice (STAT1^(-/-)^) were chosen as an *in vivo* model before progressing to gene delivery and immunogenicity studies in non-human primates. STAT1^(-/-)^ miceare highly sensitive to MPXV and the disease course in MPXV infected STAT1^(-/-)^ mice, characterized by weight loss and death by day 10 post infection—is similar to that observed in wild-type mice infected with mousepox/ectromelia virus (ECTV), the etiology agent of mousepox—and probably the best small animal model of smallpox [35,57]. Moreover it was revealed that antiviral therapy could protect mice to a degree similar to that of vaccination with Dryvax or MVA, supporting the use of STAT1^(-/-)^ mice as a reliable model to evaluate orthopoxvirus prophylactics and therapeutics [35]. STAT1^(-/-)^ mice were found to be sensitive to a wide number of pathogens due to the loss of STAT1, a key factor responsible for type 1 and 2 interferon (IFN) signaling [58–63]. BoHV-4-based vectors in wild type and immunocompromised mice behave as replicating incompetent viral vectors, showing absence of pathogenicity [31,32,64–66] and BoHV-4 replication is strongly impaired *in vitro* after the treatment of BoHV-4 infected cells with IFN-γ [67]. Considering that IFN-γ is an activator of STAT1, the potential pathogenicity of BoHV-4-based vectors in STAT1^(-/-)^ mice was a concern. However, intra peritoneal BoHV-4 inoculated STAT1^(-/-)^ mice did not show any overt clinical sign, detrimental effect or pathology correlated to challenge.

A29L, M1R, and B6R MPXV antigens were selected, as candidate antigens to be delivered by BoHV-4-based vectors, as they are orthologous to A27L, L1R and B5R Vaccinia Virus (VV) antigens respectively. A27L is a 14 kDa protein thought to be involved in VV entry events [68]. L1R is a 23–29 kDa myristoylated surface protein involved in a yet-to-be-identified post viral attachment and pre-fusion events [69]. Whereas B5R is a 42 kDa glycoprotein found on the surface of the virus [70,71] and is involved in cell surface glycosaminoglycan-mediated disruption of the viral outer membrane [72]. Further, they were shown to be able to elicit a protective immune response in mice and non-human primates when formulated in combination as a subunit vaccine consisted of purified proteins, plasmid DNA vaccines, recombinant adenovirus and alphavirus replicons [73–79]. Since the purpose of this study was to determine the capability of BoHV-4-based viral vectors to protect STAT1^(-/-)^ mice against a lethal MPXV infection, the first concern was the generation of optimized expression cassettes to be integrated into the BAC-BoHV-4-A genome that were able to efficiently express A29L, M1R and B6R antigens. Because no antibodies are available for A29L, M1R and B6R proteins, a short in-frame sequence coding for a tag peptide (gD_106_) was provided at the 3′ end of their ORFs and this allowed their expression to be monitored by western immunoblotting. Initially A29L, M1R and B6R tagged ORFs were customized under the transcriptional control of the CMV promoter; however, the only ORF to be efficiently expressed was A29L. For this reason, the CMV promoter of the M1R and B6R expression cassette was substituted with the human EF1α promoter which induced expression. The reason why the CMV promoter did not work with the ORFs of M1R and B6R has not been determined. Another interesting observation was the presence of A29L protein into the supernatant of the transfected cells despite the absence of a canonical signal peptide within the primary sequence of the protein as deduced by different signal peptide prediction software (http://www.csbio.sjtu.edu.cn/bioinf/Signal-3L/; http://www.cbs.dtu.dk/services/SignalP/; http://phobius.sbc.su.se/) [80,81]. In fact, the A29L protein analysis by SecretomeP (http://www.cbs.dtu.dk/services/SecretomeP), a sequence based method for the prediction of mammalian secretory proteins targeted to the non-classical secretory pathway, included A29L within the group of non-classical secreted proteins like fibroblast growth factors, some interleukins and galectins.

BoHV-4 is considered a virus without a clear disease association, the existence of a BoHV-4 potentially pathogenic biotype cannot be absolutely excluded when a virus is going to be exploited as a gene delivery vector. Bovine herpesvirus 4 (BoHV-4) has been most consistently associated with uterine disease in postpartum cattle and BoHV-4 infection is often identified concurrently with bacteria that cause uterine diseases [82,83]. The association between BoHV-4 infection and uterine disease has been difficult to establish. It was suggested that there may be an association with bacterial endometritis which leads to secretion of prostaglandin E2 (PGE2) and then stimulation of viral replication by PGE2, TNF-α and lipopolysaccharide (LPS)—which causes further endometrial tissue damage and inflammation [84–86]. For this reason, a putative non-pathogenic strain of BoHV-4 (BoHV-4-A) isolated from the cell milk fraction of a healthy cow whose genome was cloned as a bacterial artificial chromosome (pBAC-BoHV-4-A) [28] was used. CMV-A29LgD_106_, EF1α-M1RgD_106_ and EF1α-B6RgD_106_ were integrated into the TK locus of pBAC-BoHV-4-A, and proved to be stable through passages in *E*. *coli* SW102 and recombinant viable BoHV-4-A-CMV-A29LgD_106_ΔTK, BoHV-4-A-EF1α-M1RgD_106_ΔTK, BoHV-4-A-EF1α-B6RgD_106_ΔTK were successfully reconstituted in BEK*cre* cells. When BoHV-4-A-CMV-A29LgD_106_ΔTK, BoHV-4-A-EF1α-M1RgD_106_ΔTK and BoHV-4-A-EF1α-B6RgD_106_ΔTK were characterized in terms of replication kinetics, a reduction of replication was observed for BoHV-4-A-EF1α-B6RgD_106_ΔTK and this was attributed to a partial toxic effect induced by the abundant expression of B6RgD_106_—which was also observed in transfections of cells with EF1α-B6RgD_106_ expression cassette. This latter observation excluded a potential detrimental effect induced by the topological location of the foreign DNA in the BoHV-4 genome. Despite their replication characteristics, all three recombinant BoHV-4s abundantly expressed their transgene in infected cells.

*In vivo* protection studies determined that M1R protected against a lethal MPXV challenge. One hundred % protection was achieved when the vectors were administered twice (prime followed by booster), although the M1R expression vector was not superior to vaccination with MVA as measured by weight-loss. Protection was also afforded to mice when the vectors were administered in combination (combo) as a prime and booster. These mice experienced less weight-loss than mice vaccinated with M1R alone. This finding is surprising as the other vectors included in the combo were not protective when administered individually, although we did find protection against weight-loss when mice were treated with B6R alone. Nevertheless, our studies reveal that protection can be afforded even when a small number of mice are used. Further studies should be considered that increase the dose of vector administered to the mice. Also, since the combination of all 3 vectors gave 80% protection against mortality and morbidity, various vector permutations should be considered to elucidate the most efficacious combination and ratio of vectors.

No overt clinical-signs were observed following vaccination with the prime or booster injection, suggesting low immuno-reactivity and therefore possibly low-levels of adverse events in NHPs and humans. Although second generation live vaccines, such as ACAM2000, provide the most robust immune response, they are quite reactogenic and induce some level of morbidity in all vaccines. Furthermore, a significant portion of the human population are contraindicated to vaccination with first- and second-generation vaccines [19]. MVA is a non-replicating vaccine that has demonstrated efficacy in many animal trials. The main draw-back to MVA is its relatively low immunogenicity, meaning that booster administrations are usually required for 100% protection against morbidity and mortality [87,88]. Future studies could reveal that vectors’ studied here could be used as alternatives to MVA.

In summary, our findings have demonstrated that BoHV-4 based vectors can be used as vaccines to protect against a lethal MPXV challenge in mice. Our studies utilized STAT1^(-/-)^ mice; however, other strains have demonstrated sensitivity to MPXV, namely the CASTE/EiJ strain [49,50,89]. Future studies should consider evaluating the protection of these vectors in this strain also. This work provides a “proof-of-concept” for the BoHV-4-based vector as a potential vaccine for category A agents. Future and ongoing studies are focused on the design of BoHV-4-based vectors expressing antigens from other category A pathogens, as well as an assessment of protection in non-human primate.

## Supporting Information

S1 Fig Stability of the pBAC-BoHV-4-A-CMV-A29LgD_106_ΔTK, pBAC-BoHV-4-A-EF1α-M1RgD_106_ΔTK and pBAC-BoHV-4-A-EF1α-B6RgD_106_ΔTK plasmids in *E*. *coli* SW102 cells. (TIF)

S2 Fig Groups of mice were vaccinated with the BoHV-4 vectors containing A29R (A), M1R (B), B6R (C) or a combination of all 3 vectors (D). PBS was used as a negative control and MVA was used as a positive control for vaccination. Vaccines were administered at T = 0 days or at T = 0 days and T = 23 days (booster). Mice were monitored for weight-change from T = 0 days to T = 42 days. All mice gained or maintained weight during this period. Error bars indicate SEM. N = 5 mice per group.
(TIF)
