# Supplementary figures and images for: BoHV-4-Based Vector Single Heterologous Antigen Delivery Protects STAT1(-/-) Mice from Monkeypoxvirus Lethal Challenge
Source: PLoS Negl Trop Dis. 2015 Jun 18;9(6):e0003850. doi: 10.1371/journal.pntd.0003850 (PMC4473039; doi:10.1371/journal.pntd.0003850)

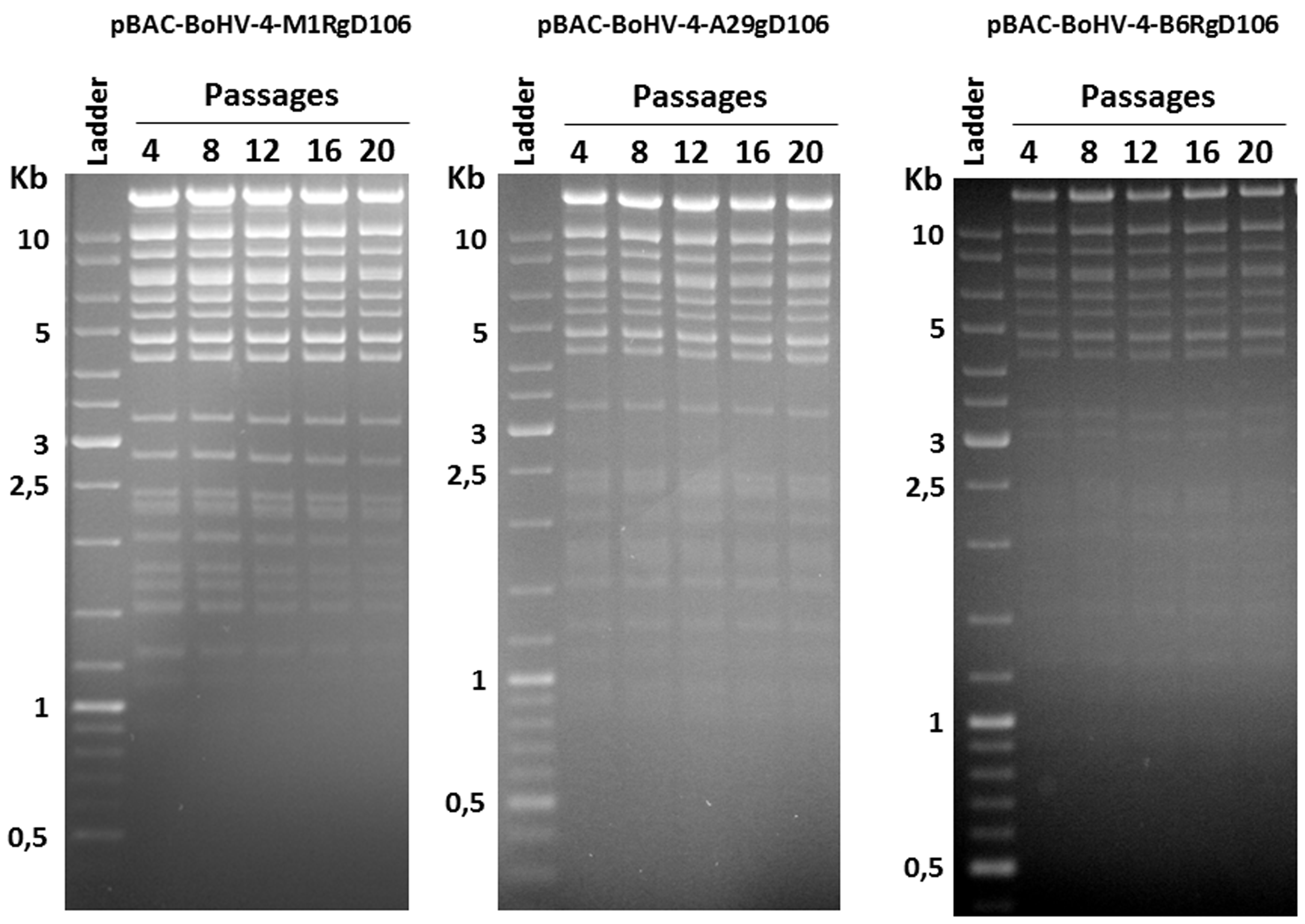

Supplement: S1 Fig — (TIF) [file pntd.0003850.s001.tif]

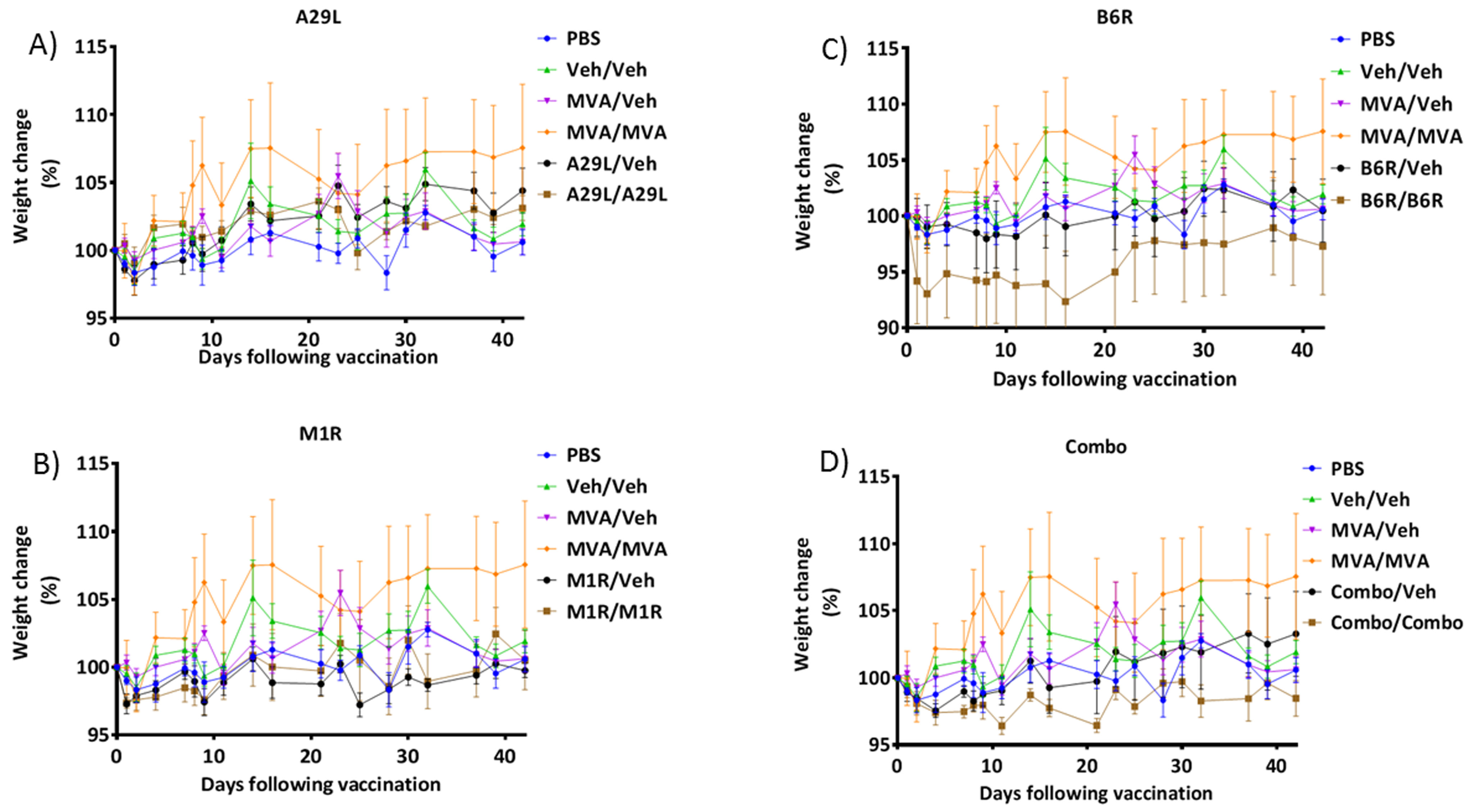

Supplement: S2 Fig — PBS was used as a negative control and MVA was used as a positive control for vaccination. Vaccines were administered at T = 0 days or at T = 0 days and T = 23 days (booster). Mice were monitored for weight-change from T = 0 days to T = 42 days. All mice gained or maintained weight during this period. Error bars indicate SEM. N = 5 mice per group. (TIF) [file pntd.0003850.s002.tif]
